# The Directionality of the Relationship Between Executive Functions and Language Skills: A Literature Review

**DOI:** 10.3389/fpsyg.2022.848696

**Published:** 2022-07-19

**Authors:** Anahita Shokrkon, Elena Nicoladis

**Affiliations:** ^1^Department of Psychology, University of Alberta, Edmonton, AB, Canada; ^2^Department of Psychology, University of British Columbia, Kelowna, BC, Canada

**Keywords:** executive functions, language, working memory, inhibition, cognitive flexibility

## Abstract

It has been demonstrated that executive functions play a significant role in different aspects of the development of children. Development of language is also one of the most important accomplishments of the preschool years, and it has been linked to many outcomes in life. Despite substantial research demonstrating the association between executive function and language development in childhood, only a handful of studies have examined the direction of the developmental pathways between EF skills and language skills, therefore little is known about how these two constructs are connected. In this review paper, we discuss three possible directional relationships between EFs and language development throughout childhood. First, we discuss how EF might affect language functioning. Next, we discuss how language functioning might affect EF. Lastly, we consider other possible relationships between EF and language. Given that children with better EF and language skills are more likely to succeed in educational settings and demonstrate greater social–emotional competencies, investigating the relationship between EF and language in the preschool period provides insight into mechanisms that have not been extensively studied. Furthermore, it could create new opportunities for designing effective and efficient interventions aimed at addressing EF and language deficits during the preschool period which could in turn influence later development.

## Introduction

Executive functions (EF) are a family of top-down mental processes involved in regulating attention, thoughts, and actions (Wiebe and Karbach, 2017). EFs enable humans to achieve goals, adjust to new life situations, and manage social interactions (Cristofori et al., 2019). EFs have been found to play a significant role in different aspects of children’s development such as planning, decision making, and problem-solving (Miyake et al., 2000; Friedman et al., 2006), academic achievement (Blair and Razza, 2007; Best et al., 2011; Richland and Burchinal, 2013; Schmitt et al., 2015), classroom learning (Blair and Razza, 2007; Liew, 2012), school readiness (Bierman et al., 2008; Shaul and Schwartz, 2014), social–emotional development (Broidy et al., 2003; Ferrier et al., 2014), physical health (Riggs et al., 2010; Moffitt et al., 2011), and making and keeping friends (Hughes and Dunn, 1998). Language development is also one of the most important accomplishments of the preschool years and has been associated with many outcomes in life. For example, it has been associated with academic achievement (Craig et al., 2003; Magnuson and Duncan, 2006), social–emotional development, and school readiness (Duncan et al., 2007; Duff et al., 2015).

Language skills and EF both undergo rapid development during preschool years and there is substantial evidence that they are associated during childhood. Although there are many correlational (Gathercole and Pickering, 2000; Carlson et al., 2005; Kuhn et al., 2014), longitudinal (Gooch et al., 2016; Pérez-Pereira et al., 2020) and intervention studies (Guttentag et al., 2014; Jones et al., 2014) showing this relationship, only few studies have focused on the direction of the developmental pathways between EF skills and language skills, therefore little is known of how these two constructs are associated. This review paper outlines three possible directional relationships between EF and language development throughout childhood. First, we discuss how EF might affect language functioning, for example children have to be able to remember what they are learning long enough to understand its meaning. So, children could learn new words through fast-mapping (a cognitive process that facilitates acquisition of new vocabulary through brief exposures to words and their referents) possibly because they remember both the word and the context that it occurred in. For instance, in an experiment when children were asked to bring the chromium tray and not the blue one (among colored trays), children were able to identify the chromium (olive green) colored tray in contrast to the others. When they were tested a week later, after a brief reminder, a majority of them had retained the association of “chromium” with the color of olive green (Carey and Bartlett, 1978). Then we discuss how language functioning might affect EF, for example, knowing words allows children to remind themselves of their current goals. Vygotsky gave an example of a child drawing a streetcar and his pencil broke. The child muttered “It’s broken” and then proceeded to draw a wrecked railroad car. So, the utterance of the word functioned as a new direction and changed the goal of the child (Packer, 2021). Finally, we discuss other possible relationships between EF and language (such as the existence of a third underlying variable or a bidirectional relationship). This could be because substantial simultaneous advancements happen in both EF and language skills during childhood and also, development of frontal lobes could impact both brain areas necessary for EF development and adjacent areas involved in language development (Bishop et al., 2014).

To provide the context for this review, we first present a brief overview of EF conceptualizations and components and, then language definitions and concepts.

### Conceptualization of EF

Although many models and conceptualizations of EF have been proposed, the most prominent model of EF has been Miyake’s and his colleagues’ (Miyake et al., 2000; Miyake and Friedman, 2012). They proposed that EF consists of three main components, inhibitory control, working memory (WM), and cognitive flexibility (Miyake et al., 2000). Together, these components contribute to higher-order EFs such as reasoning, problem-solving, and planning (Collins and Koechlin, 2012; Lunt et al., 2012). Although the structure of EF in adulthood is described by both unity and diversity, a number of studies used factor analysis to investigate the underlying structure of EF in preschool-aged children. These studies have discovered that a single, unitary factor best represents the shared variance of EF measures, rather than distinct WM, cognitive flexibility, or inhibitory control components (Wiebe et al., 2008, 2011; Willoughby et al., 2012; Fuhs et al., 2014). However, some studies in late childhood replicated findings that showed EF is composed of associated but separate components similar to adults (Miyake et al., 2000); some showed distinct WM and cognitive flexibility factors (St Clair-Thompson and Gathercole, 2006; van der Sluis et al., 2007), and a few showed distinct inhibition component (Lehto et al., 2003; Huizinga et al., 2006).

One way to reconcile these results is to posit that there is a gradual emergence of distinct EF factors over the course of childhood. Indeed, some studies have found evidence for just such gradual emergence (Shing et al., 2010; Lee et al., 2013; Gandolfi et al., 2014). Signs of differentiation emerge by 10–11 years and reach stability at 15. So, by the age of 10–11 years, the Miyake et al. (2000) model of EFs is observed (Brydges et al., 2014). The gradual differentiation of EF components is associated with progressive specialization found at the neural level. The neural networks supporting EF show increasing segregation (i.e., reduced local functional connectivity within brain regions such as the prefrontal or anterior cingulate cortex) and integration (i.e., greater distal functional connectivity between brain regions, e.g., prefrontal and parietal cortices; Fair et al., 2007). In the next part of this review, we will focus on EF components and their developmental trajectories.

### Working Memory

Working Memory (WM) refers to a mental workplace in which information is held temporarily and mentally manipulated in order to support in progress cognitive activities (Baddeley, 1992; Baddeley and Hitch, 1994). The most famous model of WM is proposed by Baddeley and Hitch (1974) and is composed of three main components of the central executive (responsible for controlling the resources, the retrieval of information from long-term memory and the scheduling of different simultaneous cognitive activities), the phonological loop (provides short-term storage for verbal information) and the visuo-spatial sketchpad (responsible for the manipulation of visual and spatial information). Another model of WM suggests a modality independent view, which does not differentiate between verbal and visual information. For instance, Engle et al. (1992) proposed attention serves to maintain or suppress information. According to their model, WM is domain-general rather than domain-specific. This model emphasizes the significance of inhibitory processes for preventing memory content from possible disruption (Engle et al., 1992).

WM is the first EF component that develops (Best and Miller, 2010). Evidence showed that infants can hold simple representations of objects in memory during the first 6 months of life (Pelphrey et al., 2004; Reznick et al., 2004). The ability to hold information in mind emerges very early and 9–12 months infants can update their WM (Diamond, 1995). By 12 months of age, the length of time that representations can be held in mind and the number of the retained items increases (Diamond and Doar, 1989). At 15 months, more complex WM abilities such as updating and manipulation of information develop, and the development continues throughout the preschool period (Gathercole, 1998; Alloway et al., 2004). During the preschool period, children gradually become capable of holding more items in mind (Gathercole, 1998) and show improvements in the phonological loop and visual–spatial sketchpad (Davis and Pratt, 1995; Gathercole, 1998, 1999; Keenan, 1998; Kemps et al., 2000; Luciana, 2003; Bull et al., 2004; Ewing-Cobbs et al., 2004; Espy and Bull, 2005). Complex WM tasks demonstrate noticeable growth in middle childhood and adolescence and continue to develop through late adolescence (Carriedo et al., 2016). WM is often measured with: A-not-B task (Piaget, 1954; Diamond, 1985), Digit Span tasks forward and backwards (Wechsler, 2008), the n-back task (Kane et al., 2007), and Corsi blocks task (Lezak, 1982).

### Inhibitory Control

Inhibitory control is the ability to control attention, behavior, and thoughts. Inhibitory control at the level of attention is the ability to selectively attend, focus on the intended stimuli and suppress attention to the unwanted stimuli (Diamond, 2012). Children with well-developed inhibitory control skills are able to stop automatic responses and use alternative responses (Diamond, 2012).

The first signs of EF appear early in life and it is known to go through substantial changes across the lifespan. Structural changes in the prefrontal cortex between the ages of two and five are associated with significant improvements in EF skills during early childhood (Zelazo and Müller, 2011). In regard to inhibition, simple forms appear within the latter half of the 1st year, showing the infant’s increasing ability to impose cognitive control over behavior, and develops throughout the toddlerhood and preschool years (Gagne and Saudino, 2010; Gagne and Hill Goldsmith, 2011). Age 3 is a pivotal age in inhibition development as children show rapid gains and improvement in tasks measuring inhibition (Carlson et al., 2004; Carlson, 2005). Improvement of inhibition continues to early adolescence (Prencipe et al., 2011).

Inhibition in young children is mostly measured with: the Delayed Response task (Goldman et al., 1970), the delay of gratification task (Mischel et al., 1973; Mischel and Moore, 1973), the whisper task (Kochanska et al., 1996), NEPSY subtests (Korkman et al., 1998), the Eriksen flanker task (Eriksen and Eriksen, 1974; Mullane et al., 2009), Simon task (Simon and Rudell, 1967), Head Toes Knees and Shoulders (HTKS) task (Burrage et al., 2008), Stroop task (Stroop, 1935; MacLeod et al., 2003), the Go/No-Go task (Godefroy and Rousseaux, 1996), and the antisaccade task (Munoz and Everling, 2004).

### Cognitive Flexibility

Cognitive flexibility (also called attention shifting) is the most complex EF component (Garon et al., 2008). It involves shifting from one “mental set” to another and allows us to think divergently, change perspective and adapt to a constantly changing environment, which is critical for academic achievement (Best et al., 2009). Children whose cognitive flexibility skills are well developed, are able to sustain and switch attention from one stimulus to another and switch between tasks when necessary (Bierman et al., 2008). Cognitive flexibility builds upon WM and inhibition (Garon et al., 2008). Cognitive flexibility develops rapidly during preschool period (Welsh et al., 1991; Paniak et al., 1996; Crone et al., 2004; Blaye et al., 2006; Dick, 2014), and continues to develop across adolescence and adulthood (Anderson, 2002).

Cognitive flexibility also has different measures such as the local/global task (Miyake et al., 2000), the dimensional change card sort (DCCS; Zelazo, 2006), the Wisconsin Card Sorting Task (WCST; Grant and Berg, 1948), nonword-repetition task (Gathercole and Baddeley, 1996) and the Number-Letter task (Rogers and Monsell, 1995).

In this review paper, I will discuss different aspects of the association between EF and language development as language has been shown to be strongly associated with EF.

### Language Development

Language is a structured system based on speech and gesture, sign, and writing that enables us to communicate with each other, express our feelings, likes, dislikes, and ideas, think about our immediate environment, define our identity, express our history and culture (Jay, 2003). Moreover, language has been shown to play an essential role in children’s school readiness and is a predictor of academic and social–emotional development (Duncan et al., 2007; Duff et al., 2015). Acquiring a language includes learning the sound pattern of the language (phonological development), learning the vocabulary (lexical development), learning the structure of the language (grammatical or morphosyntactic development) and learning how to use it to communicate (pragmatic and sociolinguistic development).

Language skills are often grouped into six categories including phonetics (the study of raw sound), phonology (the study of how sounds are used within a language), syntax (the study of word order), lexicon and semantics (the study of vocabulary and meanings), morphology (the study of words and word formation), and pragmatics (the study of language use; Harley, 2013).

The development of language starts even before children are born, as fetuses are exposed to language sounds and are learning *in utero* (May et al., 2011). The process of language development continues in infancy with prelinguistic vocalizations (Stoel-Gammon, 2001), specialization in distinguishing the speech sounds of the language in the environment (Kuhl, 2014), and a developing ability to engage in joint attention by the end of the first year (Tomasello et al., 2005). In year two, most children reach the milestones of saying their first words and combining words into multiword utterances (Fenson et al., 1994). Pronunciation, vocabulary, grammar, and pragmatics improve between ages three and five (Dickinson et al., 2003; NICHD Early Child Care Research Network, 2005). There are also major improvements as children move from home to school and start to learn new ways of using language (Hoff, 2013). In this review, I will use the term language as a description of children’s phonetics, phonology, syntax, lexicon and semantics, morphology, and pragmatic knowledge.

### Executive Function and Language

Language and EF are both multidimensional and foundational skill sets that develop rapidly in the first 5 years of children’s life (Dickinson and Tabors, 2001; Gleason and Ratner, 2009). Substantial research has established that EFs and language development during childhood are related (Bishop et al., 2014; Gooch et al., 2016). While using language and communicating, both speakers and listeners have to regulate their thoughts and actions according to their goals. Speakers need EF to choose the appropriate word among other possible choices to address the concept they want to talk about (Badre et al., 2005). Listeners need EF to organize the production of various linguistic processes to become able to interpret what other people are saying (Novick et al., 2005). Also, bilinguals probably use EF to coordinate the language they have to use for a particular interlocutor and to switch from one language to another (Ye and Zhou, 2009).

The association between EF and language is well established. Studies with both typically developing children (Müller et al., 2005; Kuhn et al., 2014) and atypical children such as children diagnosed with developmental language impairment (Henry et al., 2012; Vissers et al., 2015; Marini et al., 2020) and autism (Akbar et al., 2013) have also shown this relationship. There is also a large body of research indicating a positive association between EF and different language skills. For instance, there is evidence showing a relationship between EF and vocabulary knowledge (Carlson, 2005; Carlson et al., 2005), new vocabulary learning (Dempster and Cooney, 1982; Gathercole and Baddeley, 1993), literacy (Gathercole and Pickering, 2000), sentence reading (Lewis et al., 2006), reading comprehension (Gathercole and Pickering, 2000; Palladino et al., 2001; Booth and Boyle, 2009; Sesma et al., 2009; Booth et al., 2010; Butterfuss and Kendeou, 2018), and syntax (White et al., 2017).

There are also studies of interventions that showed success at concurrently improving children’s EF and language skills (Landry et al., 2006, 2008; Neville et al., 2013; Guttentag et al., 2014; Jones et al., 2014; Bierman et al., 2018; Marti et al., 2018). Moreover, some school-based studies showed that integrated instructional approaches (focused on language/emergent literacy skills and social–emotional competencies) could improve children’s EF and language skills (Bierman et al., 2008; Jones et al., 2014).

Therefore, understanding the developmental processes of these two important skills in young children could have significant outcomes for Early Childhood Education (ECE) to help improve the practices and provide appropriate interventions so that children could enter first grade with proper skills to become successful in school and help reduce the risks of later educational and social failure considering the importance of the first years of life in constructing the foundation for the development of these skills (Phillips and Shonkoff, 2000; Rose et al., 2008; Shonkoff, 2010; Diamond and Lee, 2011).

Despite the agreement that there are associations between EFs and language skills, there are few studies focusing on the direction of the developmental pathways between EF skills and language skills, therefore it is not exactly clear how EF and language are related. Some studies suggest simultaneous growth and relations between the EF and language skills, as significant developmental changes occur in EF and language skills of young children (Bohlmann et al., 2015). However, the theoretical and empirical evidence for the direction of the relationship (or bidirectionality) between EFs and language skills is limited and there is controversy around the timing, strength, and direction of relations (Slot and Von Suchodoletz, 2018).

In the next section, we will review the studies focusing on the directionality of the relationship between EF and language skills. First, we will discuss how EF might impact language and then how language might impact EF. Finally, we will discuss a bidirectional model suggesting that EFs and language could have a recursive relationship, and both could be important for and support the other.

### How EF Might Affect Language Functioning

Diamond (2013) proposed that EF skills could play an important role in acquisition and development of children’s language skills since EF skills help children focus on different streams of information while also monitoring errors and making decisions (Diamond, 2013). She proposed that the WM component of EF is especially important in understanding spoken or written language (Diamond, 2013). WM is required in oral language because what was said earlier is no longer physically present and WM is needed, in order to relate that to what we are hearing now. WM is also needed for understanding written language and to relate what we read earlier to what we are reading now (Diamond, 2014). Mirman and Britt (2014) also suggested that EF skills are intrinsically involved in language functioning and proposed that some aspects of EF are crucial in semantic control (the ability to flexibly access and manipulate meaningful knowledge, allowing us to focus on the relevant aspects of a concept while irrelevant information is suppressed) because as a word is heard, different lexical entries could be activated. Therefore, it is essential that specific activation of its lexical entry is enhanced (while other entries are inhibited) in order to identify the correct word. Therefore, a competent language user needs to have access to lexical representations and maintain a balance of activation and inhibition. EF could also impact linguistic abilities by increasing children’s involvement in language-developing interactions and activities (Bohlmann and Downer, 2016). For instance, children with better inhibition abilities could attend more appropriately while they are engaged in a conversation with adults, accordingly these conversations help children retain vocabulary and syntax used by the adults (Hanno and Surrain, 2019). Also, children with better cognitive flexibility might be more capable in applying the variable rules of language (Blair and Raver, 2015). For example, some words are pronounced the same but have different meanings based on the context and some conventions of language are only appropriate in specific contexts (Hanno and Surrain, 2019).

Some empirical evidence also suggests that EF skills support the development of language skills. For instance, there is some evidence showing that WM contributes to the development of vocabulary in children. Gathercole and colleagues have suggested that the phonological short-term component of WM is related to vocabulary development (Gathercole and Baddeley, 1989, 1993; Gathercole and Pickering, 2000; Gathercole, 2006). For instance, in a study they showed that the short-term phonological WM of 4-year-old children predicted vocabulary development a year later (Gathercole and Baddeley, 1989). Their research led them to the claim that phonological short-term WM (measured by a nonword-repetition task where children are required to repeat nonwords) is the basis and predictor of later vocabulary development. Even one of their studies showed that a patient with a phonological loop deficit failed to acquire the vocabulary of a new language (Baddeley et al., 1998). Other studies also demonstrated that the phonological loop of the WM construct could predict acquisition and development of vocabulary in children (Newbury et al., 2016; Verhagen and Leseman, 2016). One study examined whether WM could predict passive vocabulary development (measured with Haman and Fronczyk’s Picture Vocabulary Test – Comprehension; 2012) in 3-year-old children. Two longitudinal studies were conducted. In the first one, children’s joint attention (the ability to coordinate attention with a social partner in order to share experience) was measured at 18 months because previous studies had showed that skills (such as symbolic representational skills) developed as a result of joint attention are important predictors of later EF development (Van Hecke et al., 2012; Miller and Marcovitch, 2015). Moreover, children’s WM and vocabulary were measured at 24 months. They tested three models of the relationship between the tested variables. The only model that had a good fit to the data presented a relationship that WM significantly predicted language development of 2-year-olds, and its efficiency was conditioned by the child’s earlier competences in joint attention (Białecka-Pikul et al., 2016). In the second study, children’s WM was measured at 30 months and their passive vocabulary was measured at 24 and 36 months. They found that WM was a significant predictor of passive vocabulary at age 3. Their results indicated that WM is a significant factor in language development (Białecka-Pikul et al., 2016).

There is also evidence showing that inhibitory control abilities contribute to language acquisition (Gandolfi and Viterbori, 2020; Usai et al., 2020; Yuile and Sabbagh, 2021). For instance, McClelland et al. (2007) tested children’s inhibitory control (using the Head-to-Toes Task) and vocabulary skills (using the picture vocabulary subtest of the Woodcock Johnson Test) in the fall and spring of prekindergarten to see if gains in inhibitory control and attention skills significantly predicted growth in vocabulary. They found that children who had higher improvement in their inhibitory skills between the fall and spring terms of preschool also showed a greater improvement in their vocabulary skill when compared to children who improved less in their inhibitory skills during this time. Another longitudinal study examined the role of children’s inhibitory control skills in vocabulary knowledge of Turkish preschoolers both concurrently and subsequently 1 year later. They found that inhibitory control skills of children predicted their vocabulary knowledge at both time points and children who were better at suppressing dominant response tendencies had better vocabulary knowledge both in the present and one year later (Ekerim and Selcuk, 2018). A possible explanation is that inhibitory control skills help children direct their behaviors in a goal-oriented way by suppressing irrelevant thoughts and actions and this regulation could in turn facilitate learning from the environment (Diamond, 2013). Inhibitory control could also facilitate semantic access to words that were previously encoded by inhibiting the activation and retrieval of phonologically similar but semantically different words that in turn improves vocabulary and syntactic comprehension (Mirman and Britt, 2014). Because of the important role of inhibitory control in vocabulary development, early developmental improvements in inhibitory skills could lead to higher vocabulary knowledge and could be one of the fundamental cognitive skills explaining differences in children’s language development. Another study indicated that inhibitory control measured by Stroop test predicted grammatical ability in 5-year-old children. Their explanation was that responding correctly in both tasks requires utilizing a common cognitive capacity to inhibit irrelevant competition (Ibbotson and Kearvell-White, 2015).

Moreover, there is evidence suggesting that EF contributes to language comprehension (the ability to understand the speaker’s intent behind the message and to consider the context). Mazuka et al. (2009) proposed that immature EF, especially inhibitory functions may cause perseveration in sentence comprehension of young children that is when children are led down a wrong path and have difficulty recovering from it. For instance, when five-year-old children heard “put the frog on the napkin in the box,” they mostly thought the napkin was the main aim of the action and when they heard the last part of the sentence, “in the box” they were not able to correct their misanalysis. It is suggested that this misanalysis might be due to their incapability to inhibit the prior statement and shift their mind to consider the whole sentence. Šimleša et al. (2017) also investigated the association of EF (using dimensional change card sort, digit span task, CANTAB tasks) and language comprehension (using the The Reynell Developmental Language Scales) in preschool children. Their results indicated that the only significant predictors of language comprehension were verbal WM and inhibitory control. They suggested that language comprehension in preschoolers integrates semantic and morphosyntactic knowledge, and because they are context-dependent, inhibitory control has an important role in concentrating on a new context and inhibiting the former context and the answer that was formerly correct. They proposed that WM of preschoolers is engaged in the comprehension of long utterances (speaker’s output which might be less than a full sentence) and structurally and semantically complex sentences. The listener must decode the words, comprehend the syntax, retain the words in memory, consider the context, and have typically developed receptive vocabulary (comprehension of vocabulary) while listening to any instruction and they have to do all of this at the same time to be able to understand the whole sentence. They also suggested that verbal WM has a crucial role in the development of language comprehension because phonological WM is critical for the short-term retention of verbal information while other cognitive tasks such as words and spoken messages comprehension are happening (Baddeley et al., 1998).

Studies comparing typically developing children with children diagnosed with developmental disorders provide further evidence about the associations of language and EF. To date many studies showed that children with developmental disorders like autism (Ozonoff et al., 1991; Hughes et al., 1994; Ellis Weismer et al., 2018), Tourette syndrome (Channon et al., 2003), attention deficit hyperactivity disorder (Willcutt et al., 2005; Gau and Shang, 2010; Pellicano, 2010), children at risk of dyslexia (Gooch et al., 2016), and children with developmental language impairment (Im-Bolter et al., 2006) have weaker EF skills compared to normally developing children. For instance, a review study suggested that children and adolescents who are deaf or hard of hearing and children with developmental language impairment have deficits in EF which could even lead to social emotional problems (Smit et al., 2019). Henry et al. (2012) in a study on children with specific language impairment (SLI) found that EF skills could predict language competence and showed that EF tasks that need more English processing were harder for children who had language disabilities. The authors suggested that EF difficulties could at least partly affect the development of language and not the other way around. Another study on children with Down syndrome and autism examined the associations between EF and language skills. In children with Down syndrome, EF significantly predicted pragmatic language (i.e., social, emotional and communicative language skills) but not structural language (i.e., nonsocial language skills such as phonology, semantics, morphology and syntax). This finding could mean that the ability to interact with others in social situations using pragmatic language taps into aspects of EF such as following the conversation rules (Martin and McDonald, 2003), shifting and maintaining topics (Humphries et al., 1994), monitoring and regulating behavior (McDonald, 1993). In children with autism, EF significantly predicted pragmatic and structural language. They suggested that although children with autism might have social deficits, EF still contributed significant variability in language above and beyond social functioning (Udhnani et al., 2020).

In sum, there is substantial evidence indicating that EF contributes to language development and this contribution starts in toddlerhood and continues to adulthood. The evidence in this domain indicated the role of EF and components (WM, inhibitory control, and cognitive flexibility) in the development of grammar and vocabulary, language comprehension, and also semantic control and access.

### How Language Functioning Might Affect EF

Language acquisition has a strong impact on cognitive growth. It is suggested that communication through language allows children to improve their thinking and reasoning skills which supports the development of EFs. Therefore, exposing children to enriched language experiences (new vocabulary, verbal repetition of daily events and objects, and real-life application of words) could promote their EF skills (Tobar, 2014).

Some theories suggest that language development is more important for EF skills than the other way around. According to these theories, language skills are utilized while doing executive tasks (Winsler et al., 2009). For example, executive tasks might be facilitated by using inner speech to keep track of the instructions or talk oneself through a set of activities. This mechanism is aligned with Vygotsky’s theory which posits that language is a psychological tool that enables children to internalize self-regulation (Vygotsky, 1962). He emphasized on the importance of cultural tools, that are ways of achieving things in the world, acquired in the course of development and passed on to next generations, for the development of psychological functions such as focused attention, deliberate memory, and verbal thinking (Vygotsky, 1929/1994). He suggested two lines of development: natural development of behavior (which is closely tied with the general organic growth and the maturation of the child) and cultural development of psychological functions (the mastering of various cultural means, and the working out of new methods of reasoning). The conversion of the natural line to the cultural line of development relies on the control of behavior through language that enables the child to be free of the immediate perceptual field and to plan solutions for tasks ahead of time. Therefore, the regulatory function that adults use in interpersonal exchanges with the children is slowly internalized by the children and then used by the children themselves to regulate their behavior (Vygotsky and Luria, 1994a). The self-regulatory role of language (private speech) emerges between the ages of 2 and 5 years, which potentially fosters the development of EF during this period (Zelazo et al., 2003). Therefore, language might play an important role in consciousness and behavior control (Zelazo, 1999). Winsler et al. (2009) also provided empirical evidence supporting the claim that self-regulation develops through social interactions. The transformation of social speech to intrapersonal self-regulatory speech consists of different phases. The first phase is when external signs (such as speech sounds) help direct the attention of the child and the last phase is when the external signs are no longer needed because regulatory functions have been interiorized meaning that “the process becomes an inner-reconstructed operation” (Vygotsky and Luria, 1994b, p. 152). There are several phases between the first and the final phase. Luria further looked into the regulatory processes of speech and also identified “inner speech” as having an important effect on regulatory and planning functions. Inner speech refers to the process by which the private speech of young children, such as talking to themselves while playing, begins to accompany them in a number of cognitive tasks (Vissers et al., 2020). During early years, children seem to benefit from this kind of speech as they perform verbal labeling of the objects and actions surrounding them. This labeling process could help redirect attention between task sets and have an impact on children’s behavior and can serve as action regulation recognition of our actions (Kray et al., 2008). Also, labeling allows the word and the associated mental image (signified and signifier) to be used separately which allows language to be disengaged from direct perception, enabling more flexible cognition including EF (Toomela, 2003). It’s suggested that in children with developmental language disorder and deaf/hard of hearing children, EF deficits could be related to the mentioned property of language allowing the construction of non-sensory representations resulting from the distinction between signified and signifier (Camminga et al., 2021).

Luria also found that as children get older, they become more capable of using progressively complicated verbal commands to direct their behavior (Luria, 1959). Luria (1959, 1961) assessed the effects of labeling on a Go-NoGo task and found that 3-year-olds performed poorly in a Go-No-Go task, as older preschoolers performed better. In the Go-No-Go task, children had to press a bulb when a red light came on in go trials and had to resist pressing when a blue light came on in No-Go trials. An important finding was that when 3-year-olds were asked to use self-directed commands (such as “press”), while manually responding to the trials, they were more capable of regulating their responses. On the other hand, when 3-year-olds were asked to use self-directed commands (such as “do not press” when they had to resist responding in No-Go trials), their performance worsened. This did not happen for older children and their performance improved in both commands, as they used self-directed commands. Luria proposed that 3-year-olds are not able to direct their behavior using the meaning of the labels but can do that using the expressive and physically impulsive aspect of labels, on the other hand, older children are able to use the meaning of labels to regulate their behavior (Luria, 1959).

Moreover, Zelazo and colleagues suggested that language is an underlying precursor to the development of EF in children (Zelazo and Frye, 1998; Zelazo et al., 2003). They, in *cognitive complexity and control theory (CCC)*, suggested that children’s language and EF abilities are related and children’s ability to take specific actions to resolve conflict are dependent on their ability to use labels to create conscious representations of a problem. Moreover, language is necessary for the development and use of the embedded rule structures that helps children to solve a given problem or conflict (Zelazo and Frye, 1998). For instance, while doing a DCCS task (where participants have to sort cards one way such as by color and then are instructed to switch and sort the same cards a new way such as by shape), if a child is given a red rabbit in the color game then they should attend to the color red and act according to that, while they have to act differently sorting the 5 same cards during the shape game. “If I’m playing the colour game, and if it is red then it goes here … If I’m playing the shape game, and if it is a rabbit then it goes there.” In order to perform the task successfully, children must build embedded conditional rules that follow an “if-if-then” structure. Therefore, language is necessary for the formation and use of the embedded rule structures (the ability to formulate “if-if-then rules”) that enables children to solve a problem (Zelazo and Frye, 1998). The *Hierarchical Competing Systems Model* expanded this idea (Marcovitch and Zelazo, 2009) proposing that children’s first cognitive processes develop from a habit system that is entirely based on infants’ earlier experiences. Although with maturation, this initial habit system changes into a representational system (Marcovitch and Zelazo, 2009). Children’s language might play an active role in this transformation because the representations become stronger when children label it (Marcovitch and Zelazo, 2009). Zelazo and colleagues also posited that language has a critical role in the development of WM and goal setting which allows representation of non-present concepts, ideas, and goals. They posited that language, given its representational nature, enables the separation of action from reality, so that goal-directed behavior could be guided by action plans that are stored in WM, and not by immediate environmental stimulus (Frye et al., 1995).

Empirical evidence from the existing longitudinal studies also backs up the idea that early language abilities could predict later EF skills. Kuhn et al. (2016) examined whether vocabulary and language complexity, indicators of children’s early expressive language, predicted their EF (WM and inhibitory control) over preschool. They chose these language indicators because of their relevance to the development of EF according to CCC theory. Their results showed that the rate of a child’s change in language between 15 and 36 months was predictive of their rate of change in EF between 36 and 60 months, as well as their EF at 60 months. Their findings support the CCC theory and the idea that early language acts as a precursor to EF abilities. The proposed developmental framework by Zelazo and colleagues emphasize the use of labels as a mechanism by which the transition to representational thinking occurs (Marcovitch and Zelazo, 2009). Vallotton and Ayoub (2011) tried to extends Vygotksy’s theory regarding words as mental tools and proposed that language skills contribute to inhibitory control development. They suggested two mechanisms through which language contributes to the development of inhibitory control: (1) Children who are more talkative and use language more, have more control over their environment, are less frustrated and better regulated; and (2) children who have larger vocabularies have more symbolic representations that help them in regulating their emotions and impulses. They, in a three-wave longitudinal study with 120 children, examined the influence of expressive language skills (spoken vocabulary and talkativeness) on the development of EF. Their results showed that vocabulary at 24 months predicted EF development. Also, even after controlling for cognitive development (measures by Bayley Mental Development Index; Bayley, 1993), concurrent and prior vocabulary skills predicted children’s EF abilities. The authors concluded that even in the early stages of children’s development, words are used as a tool that could be applied to the tasks of executive functioning. Their findings that early vocabulary predicts later EF skills provides evidence consistent with Vygotsky’s (1934) theory that symbols (especially spoken words; Wertsch, 1998), are mental tools enabling humans to exert control over their thoughts, emotions, behavior and environment.

There is also evidence from studies comparing typically developing children with children having hearing problems indicating that language contributes to EF development. These studies were done because it is suggested that deafness provides a unique opportunity to disentangle these skills because in this case, language difficulties have a sensory not cognitive basis. Therefore, they may provide an opportunity to understand more about the direction of relations between different developmental skills that are not as visible when development is happening as expected. One study assessed typically developing children and deaf children, who were at risk of language delay caused by sensory difficulties, on vocabulary and non-verbal EF tasks (Botting et al., 2017). Their results showed that deaf children performed a lot weaker than normally developing children in EF tasks. The authors suggested that language is not only related to EF performance but has a role in mediating EF performance. They could not identify the reverse association suggesting that weaker performance in EF does not lead to weaker language abilities (Botting et al., 2017). A two-wave longitudinal study extending Botting et al. (2017), examined the developmental relationship between expressive vocabulary and EF in older hearing and deaf/hard-of-hearing children. Their results indicated both concurrent and longitudinal relationships between vocabulary and EF in the middle school years in both hearing and deaf/hard-of-hearing children. They found lower EF scores for deaf/hard-of-hearing children at both time points. Their findings extended the study by Botting et al. (2017) as both researchers postulated that the associations between EF and vocabulary tasks might imply the contribution of vocabulary in EF development over time and not vice versa (Jones et al., 2020).

In summary, there are studies in both typically developing and children diagnosed with developmental disorders indicating the contribution of children’s language abilities to EF performance. Most of these studies do not clearly answer the questions about whether and how emerging language, occurring during toddlerhood and the preschool period, establish a foundation on which EF skills develop. Therefore, based on the existing theoretical and empirical evidence that we will discuss in the next section, I will propose other possible relationships between EF and language in the preschool years.

### Other Possible Relationships Between EF and Language

The existing evidence could not determine the direction of the relationship between EF and language clearly, therefore, the possibility of other kinds of relationships such as the existence of a third underlying variable or a bidirectional relationship should be considered. For instance, Bishop et al. (2014) suggested that a shared genetic risk might impact development of brain systems involved in the development of both language and EF. They suggested that there may be similar underlying mechanisms involved in the development of EF and language and the connection between EF and language skills might be because they are both impacted by the same causal factors. For example, a deficit or delay in the development of frontal lobes could affect both brain areas that are crucial for EF development and adjacent areas involved in language development (Bishop et al., 2014). Also, some studies have shown that significant developmental changes occur in EF and language skills of young children (Farkas and Beron, 2004; Blair et al., 2011), that suggest simultaneous growth and reciprocal relations between the EF and language skills (Bohlmann et al., 2015). A study by Gooch et al. (2016) investigated the longitudinal associations between children’s early EF and language abilities in a sample of typically developing children and children at risk of language/literacy difficulties. They found little evidence for a significant bidirectional relationship between EF and language skills as EF and language were concurrently but not longitudinally related. Overall, the existence of a single underlying variable is not quite plausible and does not seem to be the complete answer.

The rapid development in both language and EF skills occurring during the preschool years (Farkas and Beron, 2004), combined with research that has investigated possible relationships between these two domains, propose a possible reciprocal relation in the development of language and EF. Research posits that EFs and language abilities are overlapping developmental processes as young children make great improvements in both EF and language skills simultaneously (Kaushanskaya et al., 2017). Also, there is increasing evidence that WM is related to the acquisition of vocabulary (Adams and Gathercole, 1995, 2000; Baddeley et al., 1998). Gathercole (2006) suggested that the ability to hold novel phonological forms in WM is particularly important to the formation of new words during the early stages of language development for both first and second language learning. Once children acquire some vocabulary it could help them develop regulatory abilities (Zelazo, 2015). For instance, the Iterative Reprocessing Model argues that language, specifically vocabulary and related skills (e.g., labeling), plays an important role in children’s ability to utilize higher order reasoning skills and activate intentional control of behavior (Zelazo, 2015). This is probably because rules and task instructions are usually given in formats needing a specific level of linguistic ability (Zelazo et al., 2003). Moreover, Vygotskian principles posit that vocabulary skills could help children to regulate their behaviors (Vygotsky, 1962) because verbalizations, including private speech or self-talk, probably enhance children’s ability to plan and monitor thoughts and actions that are in other words attention and inhibition abilities (Zakin, 2007). If so then attention and inhibition might follow complex language use. Then, complex language use allows further and richer development of EF skills, especially attention and inhibition. According to the Iterative Reprocessing Model, being able to develop, understand, and utilize more complex rule representations enables children to engage their WM, cognitive flexibility, and inhibitory control in overt behavior while supporting adaptability and flexibility in different environments and tasks (Zelazo, 2015). Thus, complex language use might contribute to building these fundamental skills that are important for EF development.

Recently, longitudinal data is used to study the existence of a bidirectional relationship between skills in these two domains at multiple time points. To date, there are only limited studies directly assessing the bidirectionality of the association between EF and language skills. We are aware of seven studies that tested bidirectional associations between EF and language outcomes (Fuhs and Day, 2011; Weiland et al., 2014; Bohlmann et al., 2015; Gooch et al., 2016; Daneri and Blair, 2017; Slot and Von Suchodoletz, 2018; Schmitt et al., 2019), yielding mix results. Among the mentioned studies, some found bidirectional associations between EF and language. For instance, the study by Daneri and Blair (2017) explored the bidirectional relationship between EF and expressive vocabulary in kindergarten and first grade. A total of 347 5- and 6-year-old children completed measures of EF and expressive vocabulary in the fall and spring of kindergarten and the fall of first grade. They tested expressive language as it relies on fluid abilities (individual’s ability to process information, act, and solve novel problems) of cognition that are central to EF. Path analysis showed a bidirectional relationship between these two constructs and EF in the fall of kindergarten year significantly predicted expressive vocabulary in the spring of kindergarten, and expressive vocabulary in the fall of kindergarten predicted EF in the spring. They suggested that EF skills at kindergarten are predictive of school readiness outcomes, such as expressive vocabulary (Cameron et al., 2012). Therefore, as EF skills begin to develop, they support the development of expressive language, while expressive language continues to support EF. Another recent study by Slot and Von Suchodoletz (2018) examined bidirectional associations between EFs and language skills among German preschoolers using a cross-lagged design. Language (using The Peabody Picture Vocabulary Test and, comprehension, and imitation of grammatical structures subtests of the Heidelberger Language Development Test) and EF (using The Dimensional Change Card Sort, forward digit span and copy hand movement and the pencil tapping task) development of children were measured when they entered preschool and were later compared with the data collected at the end of the school year. They found that children who had better vocabulary skills before the start of the study achieved higher scores in EF tasks during the study period. Similarly, children who started off with higher EF at wave 1 showed larger gains in their language abilities from wave one to wave two. Interestingly, their results indicated that language skills were a stronger predictor of EF development than the other way around. The authors suggested that language development is crucial for stimulating EF development and language could enhance a specific way of thinking and information processing that could promote EF skills. Bohlmann et al. (2015) investigated sequential associations between EF and English vocabulary in monolingual and bilingual Spanish–English children using cross-lagged models. They found a reciprocal relationship and simultaneous development between EF and language ability of preschoolers. The results of three waves of measurement during two years showed that there is a bidirectional association between EF and language skill development.

As mentioned, studies exploring the bidirectionality of the relationship between EF and language yielded inconsistent results and some of them failed to indicate a bidirectional relationship. Fuhs and Day (2011) explored the direction of the developmental pathways between EF and language skills in 132 children aged 4–5 years for one academic year. They measured two EF components (inhibition and cognitive flexibility) and children’s language skills once at the beginning of the school year and once at the end. Their results indicated that language skills in the fall of prekindergarten predicted EF in the spring of preschool, but that EF in the fall did not predict language ability in the spring. They suggested that the reason they could not support a bidirectional might be due to the fact that standardized measures of verbal ability were used. Another longitudinal study by Weiland et al. (2014) investigated the structural association between a latent factor representation of EF at the start of preschool and receptive vocabulary skills at the end of the preschool year using crossed-lagged structural equation models. They hypothesized that there might be a bidirectional relationship between EF skills and receptive vocabulary. Their results showed that EF skills at the beginning of preschool year significantly predicted improvements in receptive vocabulary at the end of pre-K in children but preschool-entry receptive vocabulary scores could not predict resulting EF at four years of age. They suggested that the association between verbal ability and EF could be different for receptive versus expressive vocabulary and the hypothesis that better vocabulary supports improved inner speech which in turn improves EF, might only be true for expressive vocabulary or general verbal ability. Another longitudinal study on 558 preschool children investigating the predictive relationships between vocabulary and mathematical language (a form of complex language which is related to children’s understanding of keywords and concepts in mathematics) measured in the fall of preschool and EF in the spring of the same preschool year. The results indicated that vocabulary and mathematical language scores at preschool entry was a significant predictor of EF scores in spring. They also examined the bidirectional relationships between language and EF and found that vocabulary in the fall did not predict EF in the spring; on the other hand, EF in fall predicted vocabulary in the spring. Moreover, mathematical language predicted EF and also EF significantly predicted mathematical language in spring (Schmitt et al., 2019).

In sum, there is evidence that EF influences language and there is also some evidence for vice versa. Therefore, it seems likely that both directions of causality are at least somewhat true. Also, the possibility of other kinds of relationships such as a relationship where a third underlying variable exists or a bidirectional one, should be considered. Existing evidence shows that there are simultaneous improvements in both EF and language skills in young children and a delay in the development of frontal lobe could affect both important brain areas for EF and language development. Also, WM is important for the acquisition of vocabulary, especially for the formation of new words. Acquiring some vocabulary could lead to the development of regulatory abilities that could be because rules and task instructions are usually given in formats needing a specific level of linguistic ability. Then, planning and monitoring thoughts and actions, which are attention and inhibition abilities, would be enhanced and that might follow complex language use. Then, complex language use allows further development of EF skills, especially attention and inhibition.

## Discussion

Considering that children with better EF and language skills are more likely to succeed in educational settings and demonstrate more social–emotional competencies (Blair et al., 2011; Wanless et al., 2011; Weiland et al., 2014), establishing the relationship between EF and language in the preschool period could provide insight into mechanisms that have not been widely studied. Also, it could create new opportunities for designing effective and efficient interventions targeting EF and language deficits during the preschool period which in turn could affect later development.

Although there is clear evidence showing that language and EF are related, the current existing studies could not determine the clear direction (or bidirectionality) between EF and language development as the existing literature examining this relationship has some limitations that should be addressed in the future studies. Future studies should consider using more suitable study designs and expanding their sample size. Many of the existing studies are cross-sectional and correlational studies that could only show a concurrent relationship. In order to confirm the bidirectionality of the relationship longitudinal studies are needed and the current number of longitudinal studies is minimal and their results are very inconsistent. Also, most studies in this domain assessed EF based on a single measure and measured only one domain of language (mostly vocabulary) thus the extent to which the different domains of language are differentially associated with EF is mostly unknown. Therefore, as there are inconsistencies in the line of research investigating the directionality of the relationship between EF and language skills, future longitudinal studies with more participants should investigate the relationship between different aspects of language (syntax, semantics, and pragmatics) and different components of EF (WM, inhibition, and cognitive flexibility).

Moreover, future studies should consider including non-verbal tasks to measure EF. Most of the current studies investigating the relationship between EF and language skills have used verbal tasks to measure EF. For instance, for the dimensional change card sort (DCCS) task, the examiner uses verbal prompts to present cues and instructions and using verbal commands for investigating the relationship between language and cognitive functions is not effective.

## Author Contributions

AS: drafting the article. EN: supervision. All authors contributed to the article and approved the submitted version.

## Funding

This study received funding from a Discovery grant (#2018-04978) from the Natural Sciences and Engineering Research Council of Canada to EN.

## Conflict of Interest

The authors declare that the research was conducted in the absence of any commercial or financial relationships that could be construed as a potential conflict of interest.

## Publisher’s Note

All claims expressed in this article are solely those of the authors and do not necessarily represent those of their affiliated organizations, or those of the publisher, the editors and the reviewers. Any product that may be evaluated in this article, or claim that may be made by its manufacturer, is not guaranteed or endorsed by the publisher.
